# Comparison of the effects of photodynamic therapy, intravitreal ranibizumab and combination for polypoidal choroidal vasculopathy under 1 + PRN regimen

**DOI:** 10.1186/s12886-018-0801-7

**Published:** 2018-06-20

**Authors:** Kunbei Lai, Ying Li, Lijun Zhou, Xiaojin Zhong, Chuangxin Huang, Fabao Xu, Lin Lu, Jian Ge, Chenjin Jin

**Affiliations:** State Key Laboratory of Ophthalmology, Zhongshan Ophthalmic Center, Sun Yat-sen University, 54 South Xianlie Road, Guangzhou, 510060 China

**Keywords:** Polypoidal choroidal vasculopathy, Photodynamic therapy, Ranibizumab, Combination therapy, Cost-benefit

## Abstract

**Background:**

The optimal treatment for polypoidal choroidal vasculopathy (PCV) is still under debate. Little knowledge is known about the treatment effect of “1+pro re nata(PRN)” treatment regimen for PCV. The aim of this study was to compare the outcomes of photodynamic therapy (PDT), intravitreal ranibizumab injection (IVR) and combination therapy under the “1 + PRN” treatment regimen for PCV.

**Methods:**

Fifty-seven eyes of 57 patients completed the 12 months’ follow-up in this prospective study. The patients in the PDT arm(*n* = 23), ranibizumab arm(*n* = 18), or combination arm(*n* = 16) underwent a session of PDT, IVR or combination of both at baseline followed by additional IVR as needed. Mean change of logarithm of the minimal angle of resolution (logMAR) visual acuity (VA), central foveal thickness (CFT) and the regression rate of polyps were evaluated. Cost-benefit analysis was also performed.

**Results:**

At Month 12, the mean logMAR VA improved from 0.90 ± 0.52 to 0.75 ± 0.57 in the PDT group (*P* < 0.05), from 0.96 ± 0.58 to 0.77 ± 0.41 in the IVR group (*P* < 0.05), and from 0.94 ± 0.55 to 0.72 ± 0.44 in the combination group (*P* < 0.05), respectively. The CFT decreased from 478.04 ± 156.70 μm, 527.5 ± 195.90 μm, and 522.63 ± 288.40 μm at the baseline to 366.43 ± 148.28 μm, 373.17 ± 134.88 μm and 328.44 ± 103.25 in the PDT group (*P* < 0.05), IVR group (*P* < 0.01), and the combination group (*P* < 0.05), respectively. However, no statistical difference was found between groups (*P* > 0.05). PDT treatment (60.87%) was superior to the IVR therapy (22.22%) in achieving complete regression of polyps (*P* < 0.05). Cost-benefit analysis showed that IVR treatment cost the least money for improving per 0.1logMAR units and the combination therapy demanded the least money for reducing per 100 μm of CFT.

**Conclusions:**

PDT, IVR and the combination therapy have similar efficacy in the VA improvement as well as the reduction of CFT under the “1 + PRN” treatment regimen.

**Trial registration:**

Current Controlled Trials NCT03459144. Registered retrospectively on March 2, 2018.

## Background

Polypoidal choroidal vasculopathy (PCV), characterized by the polypoidal lesions and the branching vascular network visualized on indocyanine green angiography (ICGA), is considered as a subtype of wet age-related macular degeneration (AMD) which is the leading cause of irreversible sight-threatening diseases among the older adults in developed countries [1–4]. However, patients with PCV tend to be much younger, more likely Asians, and usually present with hemorrhagic pigment epithelial detachment (PED) [2]. Besides, the genetics, clinical features, prevalence, and the nature history of PCV are different from those of AMD [2, 5]. Therefore, some ophthalmologists consider that PCV is a distinct clinical entity differing from AMD [2, 6, 7].

Although the treatment regimens for AMD have been well-established, the optimal treatment for PCV is still under debate [8]. In fact, “1 + PRN” treatment schedule (1 + pro re nata, namely one intravitreal ranibizumab injection at baseline followed by as-needed reinjection) is now widely used for treating PCV patients in many provinces in China. However, there is still lack of knowledge about the efficacy of “1 + PRN” treatment regime for the PCV patients till now, especially for the Chinese mainland patients. This study aimed to compare the treatment outcomes of photodynamic therapy (PDT), intravitreal ranibizumab injection (IVR) and combination therapy under the “1 + PRN” treatment regimen for Chinese mainland patients with macula-involved PCV.

## Methods

### Patients

This study was a single center report of a multicenter, prospective, interventional clinical study which has not been published yet. Fifty-seven eyes of 57 patients with macula-involved PCV who were treated at Zhongshan Ophthalmic Center of Sun Yat-sen University from December 2012 through July 2015 were randomly assigned to each treatment group and completed the 12 months’ follow-up in this prospective study. This single center study has the same inclusion criteria and exclusion criteria with the multicenter study. The inclusion criteria were: 1) active macula-involved polypoidal lesions evidenced by ICGA; 2) greatest linear dimension of 5400 μm or less assessed by ICGA; 3) follow-up of at least 12 months. The exclusion criteria were: 1) any other ocular disease, such as ocular trauma, glaucoma, uveitis, diabetic retinopathy, angioid streaks, pathologic myopia, or presumed ocular histoplasmosis syndrome; 2) any systemic contraindication to the PDT, IVR, sodium fluorescein, or indocyanine green dyes; 4) any severe uncontrolled systemic disease, such as uncontrolled hypertention, coronary heart disease, liver failure, or kidney failure. This study was approved by the Institutional Review Board of Sun Yat-sen University and conducted in accordance with the tenets of the Declaration of Helsinki. Written informed consent was obtained after all the subjects received detailed explanations for the study protocol. The study was registered on the https://www.clinicaltrials.gov/ct2/results?cond=&term=NCT03459144&cntry=&state=&city=&dist=/ (trial registration number: NCT03459144).

### Treatment

Fifty-seven eyes of 57 patients with macula-involved PCV completed the 12 months’ follow-up and were analyzed in this study. All the patients in each treatment groups were treated under the “1 + PRN” treatment regimens: for the “1 + PRN” PDT monotherapy group, patients (*n* = 23) underwent a session of PDT with verteporfin (Visudyne®; Novartis International AG, Basel, Switzerland) at baseline followed by additional PDT as needed; patients in the “1 + PRN” IVR monotherapy group (*n* = 18) received a single intravitreal injection of ranibizumab (0.5 mg Lucentis®; Genentech, South San Francisco, CA) at baseline and additional IVR was performed when needed; and patients in the “1 + PRN” combination therapy group (*n* = 16), the patients underwent a session of verteporfin PDT followed by a single IVR 72 h after the PDT treatment at baseline and only additional IVR was given to the patient pro re nata. The PDT was performed according to the standard TAP guidelines [9] and the intravitreal injections were performed under standard sterile conditions, as described previously [10]. Retreatment were conducted if any of the following occurred according to the criteria of PrONTO Study [11]: 1) VA loss of at least 0.1 logMAR unit (equivalent to 5 letters of the Early Treatment Diabetic Retinopathy Study (ETDRS) chart together with optical coherence tomography (OCT) evidence of fluid in the macula; 2) an increase of central foveal thickness (CFT) of more than 100 μm based on OCT images; 3) enlargement of a PED; 4) new macular hemorrhage; 5) newly formed PCV; 6) evidence of persistent fluid on OCT 1 month after the previous injection. Specially, the interval of two PDT treatments must be at least 3 months and the interval of two IVR treatments must be at least 1 month.

### Assessment

Best-corrected visual acuity (BCVA), slit-lamp examination, tonometry, funduscopy, and OCT (Spectralis HRA + OCT; Heidelberg Engineering, Germany) were performed at baseline and at the follow-up of month 1, 2, 3, 6, 9, and 12 routinely. Fundus fluorescein angiography (FFA) and ICGA were performed at baseline and month 12 of follow-up. Additional follow-up visit and examinations including OCT, FFA, and ICGA would be arranged if the patients had severe vision loss or when it was necessary to assess and confirm treatment plan for patients with recurrent or when the surgery performer considered it was necessary to increase the frequency of inspections for the patient to avoid the severe vision loss or complications.

The BCVA was measured using Snellen chart and was converted the values to logarithm of the minimal angle of resolution (logMAR) equivalent which could be used for statistical analysis directly. The CFT was determined by the average foveal thickness of the vertical and horizontal foveal thickness which were measured manually from the inner retinal surface to the retinal pigment epithelium (RPE) line, as described previously [12]. Besides, in this single center report, we calculated and analyzed some other parameters which were not included in the multicenter clinical trial, such as the proportion of patients who had complete regression of polyps and the cost-benefit analysis.

### Statistical analysis

The difference in mean changes of logMAR VA and CFT was investigated using the one-way repeated-measures analysis of variance (ANOVA). Independent variables between two groups (such as the number of injections between the IVR group and the combination group) were used the Mann-Whitney U test and the categorical data were analyzed using the chi-square test. All the statistical analysis was performed using SPSS 13.0 software (SPSS, Chicago, Illinois, USA). A *P* value less than 0.05 was considered statistically significant.

## Results

### Baseline characteristics of the patients

A total 57 eyes of 57 patients completed the 12 months’ follow-up in this study. The baseline clinical characteristics of all the patients were shown in Table 1. No substantial imbalances in the demographic or ocular characteristics of the patients among the three groups was found at baseline (*P*>0.05).

**Table 1:** The baseline clinical characteristics of the patients with PCV

	Ranibizumab (*n* = 18)	Verteporfin PDT (*n* = 23)	Verteporfin PDT + Ranibizumab (*n* = 16)	*P* value
Mean age of onset, mean ± SD	64.67 ± 8.52	60.52 ± 7.77	61.06 ± 9.12	0.26
Gender				
Male, no. (%)	12 (66.67)	14 (60.87)	10 (62.50)	0.93
Female, no. (%)	6 (33.33)	9 (39.13)	6 (37.50)	0.93
BCVA (logMAR units), mean ± SD	0.96 ± 0.58	0.90 ± 0.56	0.94 ± 0.55	0.93
GLD (μm), mean ± SD	2821.03 ± 1232.41	2370.56 ± 1311.79	2175.84 ± 1181.34	0.25
CFT (μm), mean ± SD	527.50 ± 195.90	478.04 ± 156.70	522.63 ± 288.40	0.87
Mean number of polyps per patient	5.39 ± 2.79	5.35 ± 2.25	5.06 ± 3.80	0.94
Presence of PED, no. (%)	15 (83.33)	21 (91.30)	15 (93.75)	0.57
Presence of leakage, no. (%)	18 (100)	22 (95.65)	16 (100)	0.59

*PDT* photodynamic therapy, *SD* standard deviation, *BCVA* best-corrected visual acuity, *logMAR* logarithm of minimal angle of resolution, *GLD* greatest linear dimension of lesion, *CFT* central foveal thickness, *PED* pigment epithelial detachment

### Mean numbers of treatments

The mean (±SD) numbers of the intravitreal injections of ranibizumab were 3.83 ± 1.20 and 2.38 ± 1.09 in the IVR monotherapy group and in the combination group during the 12-month follow-up (including the loading phase), respectively. The patients received 1.74 ± 0.69 sections of PDT treatments on average in the PDT monotherapy group during the 12-month follow-up (including the loading phase). There was statically difference for the numbers of injections between the IVR group and the combination group (*P* < 0.01).

### Best-corrected visual acuity

**Fig. 1:**
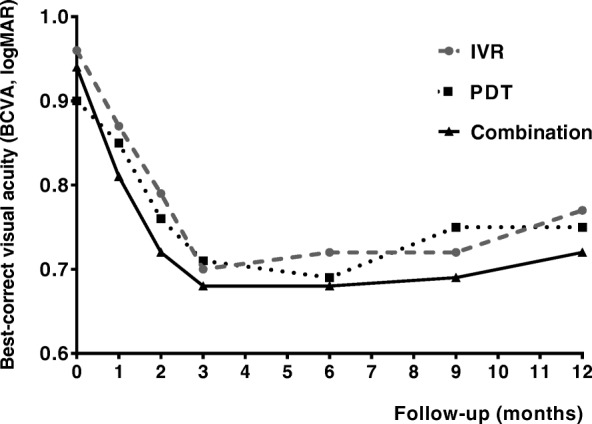
The changes of the mean logarithm of the minimum angle of resolution (logMAR) visual acuity (VA) during the 12-month follow-up. In the PDT group, there were significant differences for the logMAR VA at each time-point compared with the baseline except month 1 and month 2 (*P* > 0.05 for month 1 and month 2, *P* < 0.05 for all the other time-points). In both the IVR group and the combination group, significant differences were found at each time-point compared with the baseline except month 1(*P* > 0.05 for month 1, *P* < 0.05 for all the other time-points)

**Fig. 2:**
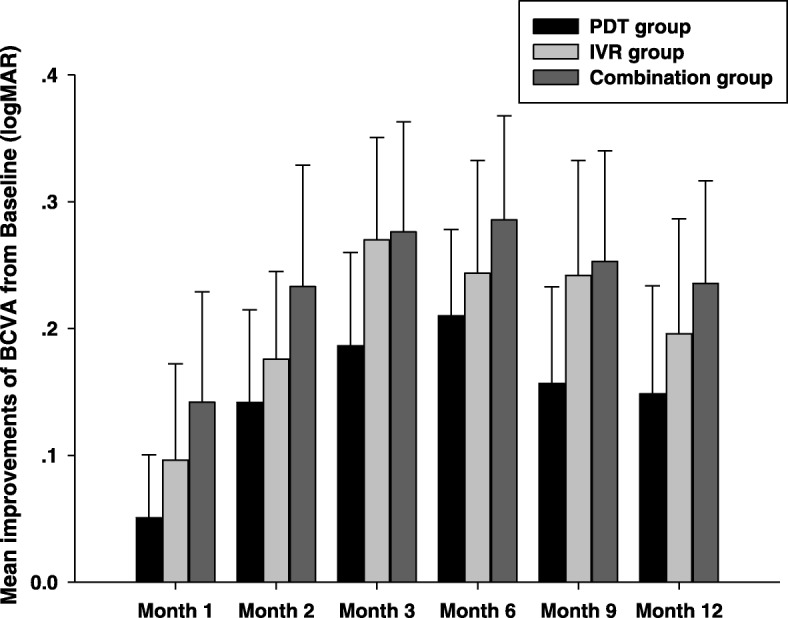
Mean improvements of best corrected visual acuity (BCVA) in logMAR VA during the 12-month follow-up. Although no statistical difference was found for the improvements of the logMAR VA between any two groups at any follow-up time-point (*P* > 0.05), a trend that combination group might have greater improvements compared with PDT or IVR monotherapy group could be seen from the histogram. Data were expressed as mean ± SEM

The changes of logMAR VA during the 12-month follow-up in each group were shown in Fig. 1. For the IVR monotherapy group, the baseline logMAR VA was 0.96 ± 0.58, which improved to 0.87 ± 0.61, 0.79 ± 0.54, 0.70 ± 0.51, 0.72 ± 0.45, 0.72 ± 0.43, 0.77 ± 0.41 at month 1, 2, 3, 6,9, and 12, respectively. There were significant differences for the logMAR VA at each time-point compared with the baseline except month 1 (*P* > 0.05 for month 1 compared with baseline; *P* < 0.05 for all other time-point compared with baseline). In the PDT monotherapy group, the mean logMAR VA significantly increased from 0.90 ± 0.52 at the baseline to 0.85 ± 0.55 (month 1, *P* > 0.05), 0.76 ± 0.52 (month 2, *P* > 0.05), 0.71 ± 0.55 (month 3, *P* < 0.05), 0.69 ± 0.54 (month 6, *P* < 0.05), 0.75 ± 0.60 (month 9, *P* < 0.05), and 0.75 ± 0.57 (month 12, *P* < 0.05), respectively (all compared with the baseline). And in the combination group, the mean logMAR VA significantly increased from 0.94 ± 0.55 at the baseline to 0.81 ± 0.43, 0.72 ± 0.44, 0.68 ± 0.45, 0.68 ± 0.43, 0.69 ± 0.42, 0.72 ± 0.44 at follow-up of month 1, 2, 3, 6,9 and 12, respectively (*P* > 0.05 for month 1 and *P* < 0.05 for all the other time-points compared with the baseline). However, no statistical difference was found for the changes of the logMAR VA between groups (*F* = 0.048, *P* > 0.05). Mean improvements of BCVA from baseline in PDT monotherapy group (0.05 ± 0.05, 0.14 ± 0.07, 0.19 ± 0.07, 0.21 ± 0.07, 0.15 ± 0.08, and 0.15 ± 0.09 at each time-point of follow-up, respectively), IVR monotherapy group (0.10 ± 0.08, 0.18 ± 0.07, 0.27 ± 0.08, 0.24 ± .09, 0.24 ± .09, and 0.20 ± 0.09 at each time-point of follow-up, respectively), and combination group (0.14 ± 0.09, 0.23 ± 0.10, 0.28 ± 0.09, 0.29 ± 0.08, 0.25 ± 0.09, and 0.24 ± 0.08 at each time-point of follow-up, respectively) during the 12-month follow-up were shown in Fig. 2. Although no statistical difference was found for the improvements of the logMAR VA between any two groups at any follow-up time-point (*P* > 0.05), a trend that combination group might have greater improvements compared with PDT or IVR monotherapy group could be seen from the histogram. Specially, to compare the ability of three different treatment regimes on preserving or improving the BCVA of the patients, we calculated the proportion of patients who gained more than 0.2 logMAR units, demonstrated no change (change less than 0.2 logMAR units), or lost more than 0.2 logMAR units at month 12. Our data showed that the proportion of patients who gained, no change, or lost more than 0.2 logMAR units were 39.89, 50.00 and 11.11% in the IVR group, 30.43, 56.52 and 13.04% in the PDT group, and 31.25, 56.25 and 12.50% in the combination group, respectively. However, no statistical difference was found between any two groups (*P* > 0.05).

### Central foveal thickness

**Fig. 3:**
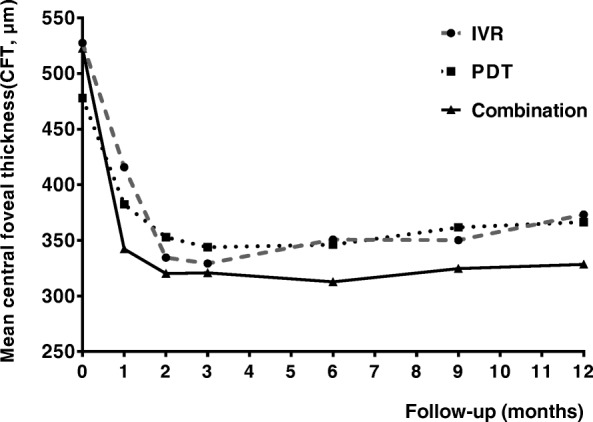
Time course of the central foveal thickness (CFT) during the 12-month follow-up. All the patients experienced a statistically significant decreasement of CFT at each time-point compared with the baseline in each group (*P* < 0.05 for all time-points in each group). However, no statistical difference was found between any two groups (*P* > 0.05)

**Fig. 4:**
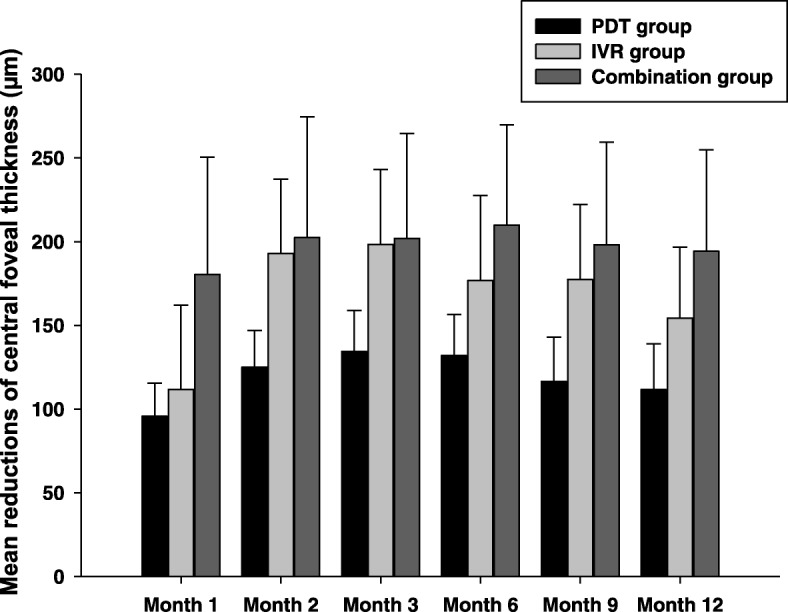
Mean reductions of central foveal thickness (CFT) from baseline during the 12-month follow-up. Although no statistical difference was found for the reductions of CFT between any two groups at any follow-up time-point (*P* > 0.05), a trend that combination group might have greater reduction when compared with PDT or IVR monotherapy group could be seen from the histogram. Data were expressed as mean ± SEM

The CFT was the average value of the vertical and horizontal foveal thickness which were measured manually from the inner retinal surface to the RPE line. Figure 3 showed the changes of the mean CFT during the 12-month follow-up in each group. The mean CFT decreased significantly from 527.5 ± 195.90 μm at the baseline to 415.78 ± 205.93 μm (*P* < 0.05), 334.5 ± 126.99 μm (*P* < 0.01), 329.17 ± 106.27 μm (*P* < 0.01), 350.67 ± 130.6 μm, 350.11 ± 115.89 μm (*P* < 0.01), and 373.17 ± 134.88 μm (*P* < 0.01) in the IVR group at the time-point of month 1, 2, 3, 6, 9, and 12, respectively. In the PDT monotherapy group, the CFT at baseline was 478.04 ± 156.70 μm, which decreased significantly to 382.35 ± 145.68 μm (month 1, *P* < 0.01), 352.91 ± 140.81 μm (month 2, *P* < 0.01), 343.74 ± 144.79 μm (month 3, *P* < 0.01), 346.09 ± 144.79 μm (month 6, *P* < 0.01), 361.65 ± 154.18 μm (month 9, *P* < 0.01), and 366.43 ± 148.28 μm (month 12, *P* < 0.05), respectively. It was also noted that the mean CFT decreased significantly from 522.63 ± 288.40 μm at the baseline to 342.13 ± 106.82 μm, 320.13 ± 106.94 μm, 320.75 ± 112.60 μm, 312.75 ± 89.15 μm, 324.56 ± 94.77 μm, and 328.44 ± 103.25 μm at above time-points respectively in the combination group (all *P* < 0.05 at each time-point of follow-up). Although significant difference in the mean CFT was found between the baseline and every time-point of follow-up in each group, no significant difference was found between any two groups (*F* = 0.029, *P* = 0.866). Mean reductions of CFT in PDT monotherapy group (95.70 ± 19.70, 125.13 ± 21.78, 134.30 ± 24.60, 131.96 ± 24.50, 116.39 ± 26.51, 111.61 ± 27.40 at each time-point of follow-up, respectively), IVR monotherapy group (111.72 ± 50.28, 193.00 ± 44.40, 198.33 ± 44.61, 176.83 ± 50.71, 177.39 ± 44.78, 154.33 ± 42.41 at each time-point of follow-up, respectively), and combination group (180.50 ± 70.01, 202.50 ± 72.14, 201.88 ± 62.77, 209.88 ± 59.89, 198.06 ± 61.39, 194.19 ± 60.58 at each time-point of follow-up, respectively) from baseline to Month 12 were shown in Fig. 4. Although no statistical difference was found for the reductions of CFT between any two groups at any follow-up time-point (*P* > 0.05), a trend that combination group might have greater reduction of CFT when compared with PDT or IVR monotherapy group could be seen from the histogram.

### Regression of polyps

In this prospective study, we not only calculated the proportion of patients who had complete regression of polyps, but also compared the proportion of partial regression of polyps and no regression of polyp after 12-months treatment in each group, respectively. In the IVR monotherapy group, 4 of 18 (22.22%) eyes had their polyps completely regressed, 4 of 18 (22.22%) eyes had partial regression of polyps, and 10 of 18 eyes (55.56%) had no response to the anti-vascular endothelial growth factor(VEGF) therapy at month 12 time-point. The proportion of patients whose polyps completely or partially regressed was higher in the combination group: 6 of 16 (37.5%) eyes had complete regression of polyps, 7 of 16 (43.75%) eyes had partial regression, and 3 of 16 (18.75%) eyes had no response to the treatment. The proportion of complete regression of polyps was 14 of 23 (60.87%) in the PDT group with partial regression rate of 17.39% (4 of 23 eyes) and no response rate of 21.74% (5 of 23 eyes). Statistical analysis showed that there was significant difference for the proportion of complete regression of polyps between the PDT group and IVR group (*P* < 0.05).

### Cost-benefit analysis

Table 2 showed the cost-benefit of the treatment regimes of the IVR monotherapy, the verteporfin PDT monotherapy and the combination therapy at month 12. The total cost of per treatment for IVR and PDT in our hospital were 1186.4 and 2515.2 US dollars, respectively. We calculated the average money which was spent for improving per 0.1 logMAR units in each group but no statistical difference was found between groups (*F* = 2.18, *P* > 0.05). However, we could see the trend that the patients in the IVR monotherapy group spent the least money in achieving improvement of per 0.1 logMAR units. There was statistically significant difference for the money which spent for reducing per 100 μm CFT between the IVR group and the PDT group (*P* < 0.05), as well as between the PDT group and the combination group (*P* < 0.01), although no statistical difference was detected between the IVR group and the combination group (*P* > 0.05).

**Table 2:** Cost-benefit analysis for different treatment regimes (Month 12)

	Ranibizumab	Verteporfin PDT	Verteporfin PDT + Ranibizumab
Mean number of IVR	3.83	0	2.38
Mean number of PDT	0	1.74	1.00
Mean BCVA improvement (logMAR unit)	0.19	0.15	0.22
Mean reduction of CFT (μm)	154.33	111.61	194.19
Money for per 0.1 logMAR units improvement	2319.53	2917.63	2426.74
Money for per 100 μm reduction of CFT	2944.28	3921.20	2749.28

### Safety

One patient from the PDT group (1 out of 23, 4.35%) and one patient from the combination group (1 out of 16, 6.25%) experienced retinal hemorrhage and subsequent visual impairment which were suspected to be related to the PDT treatment. One patient in the IVR group (1 out of 18, 5.56%) had the intraocular pressure elevation which was thought to be caused by the surgery procedure. No other serious ocular adverse event such as endophthalmitis, uveitis, lens damage, or retinal pigment epithelium tear was seen in this study. For the systemic side effects, transaminase elevation was seen in a patient (1 out of 18, 5.56%) who has a history of chronic hepatitis B two months after the intravitreal injection of ranibizumab in the IVR group. Besides, a lacunar cerebral infarction was diagnosed 2 months after the combination therapy in a patient (1 out of 16, 6.25%) who has a 10 years’ history of hypertension in the combination group. However, there is no direct evidence certificating the above systemic side effects were caused by the IVR or the PDT treatment.

## Discussion

Verteporfin PDT, which is the first effective and safe treatment for wet AMD [3], has also been reported to be a favorable treatment for PCV patients [13, 14]. Some clinical studies has documented that a single treatment of verteporfin PDT could preserve and improve the visual acuity in more than 80% patients [15–17]. The EVEREST study was the first randomized controlled trial evaluating the treatment outcomes of verteporfin PDT with or without ranibizumab versus ranibizumab monotherapy and their data showed that the PDT treatment had a superior efficacy in the closure of PCV polyps compared with the ranibizumab monotherapy although they failed to established the differences in visual acuity or in the changes of central retinal thickness [13]. The LAPTOP study, a multicentered randomized controlled trial aimed to compare the effects of PDT and intravitreal ranibizumab in patients with PCV, demonstrated that both the proportion of patients gaining more than 0.2 logMAR units and the mean gaining of logMAR VA were greater in the IVR arm compared with the PDT arm at month 12 [1]. So, it seems that anti-VEGF therapy is superior in preserving and improving the visual acuity of PCV patients while the PDT treatment might be more effective in the closure of polyps. Recently, EVERESTII study demonstrated that combination therapy of ranibizumab plus verteporfin PDT was superior to “3 + PRN” ranibizumab monotherapy in BCVA and superior in complete polyp regression [18]. However, the most effective treatment modality with the least side effects for PCV patients has not yet been completely established.

It is noteworthy that many recent PCV studies including the EVEREST study and the LAPTOP study take the “3 + PRN” treatment regime in the ranibizumab arm [1, 13, 18–21], namely 3 monthly intravitreal injections of 0.5 mg ranibizumab at the initial treatment followed by as needed (pro re nata) repeat treatments. This “3 + PRN” treatment regime for wet AMD is well established basing on the fact that patients gain their most of VA improvement during the first 3 months followed by less than improvement of 2 letters for the rest 9 months during the first year [22–24]. However, as we know, PCV is an entity differing from AMD [2, 6, 7], therefore, whether “3 + PRN” treatment regime of IVR is necessary or over-treating for PCV patients is still unclear. Three times of monthly intravitreal injections of ranibizumab for PCV at the loading phase are huge economic burdens for Chinese PCV patients, especially for those who living in the rural areas of China. In fact, nowadays, the practice of “1 + PRN” for treating PCV is prevalent in many regions of China. However, there is still little knowledge about the treatment outcome of IVR under the “1 + PRN” treatment regime. Our data showed that one initial treatment followed by an as-needed retreatment (1 + PRN) regime in the PDT arm, the IVR arm and the combination therapy arm all had improved BCVA (Figs. 1 and 2) and reduced the central foveal thickness (Figs. 3 and 4) compared with the baseline throughout 12-month follow-up. However, no significant difference was found for neither the improvement of BCVA nor for the reduction of CFT between any groups at any time-point, suggesting that both the IVR and the combination therapy had similar treatment efficacy compared with verteporfin PDT treatment under the “1 + PRN” treatment regimen. When comparing the mean numbers of intravitreal injections between the IVR arm and the combination therapy arm, we did find fewer numbers of IVR in the combination therapy arm than that in the IVR arm (*P* < 0.01), suggesting that a combination of PDT and IVR at initial treatment could decrease the total numbers of intravitreal injections. In addition, we calculated the proportion of patients who gained more than 0.2 logMAR units, demonstrated no change (change less than 0.2 logMAR units), or lost more than 0.2 logMAR units at month 12 and found that the proportion were 30.43, 56.52 and 13.04% in the PDT group, 39.89, 50.00 and 11.11% in the IVR group, and 31.25, 56.25 and 12.50% in the combination group, respectively. However, no statistical difference was detected for the proportion between any two groups (*P* > 0.05), suggesting that these three treatment modalities had similar ability of preserving or improving the visual acuity of the PCV patients.

A growing body of evidence has shown that verteporfin PDT was superior to anti-VEGF therapy in achieving complete regression of polyps. In the EVEREST study, the proportion of patients with complete regression of polyps at month 6 was 77.8% in the verteporfin PDT combined with ranibizumab group and 71.4% in the PDT monotherapy group, which were statistically significantly higher than the ranibizumab monotherapy group [13]. Other studies had the similar outcomes, with complete polyp regression rate varied from 68.4 to 100% in the patients treated with PDT, and varied from 16.67 to 60.6% in the patients treated with IVR [10, 25–27]. In our study, the proportion of complete regression of polyps was 60.87, 22.22 and 37.5% in the PDT monotherapy group, the ranibizumab monotherapy group and the combination therapy group, respectively. Interestingly, the complete regression rate in the combination therapy group was much lower than that of the PDT monotherapy group in our study. The possible reason for it was that patients in the combination therapy group in our study underwent only one section of PDT throughout the 12-months follow-up, which might not so efficiency in achieving complete regression of polyps. The regimen that only one section of PDT in the combination therapy group in our study also explained the reason why our results differed from other studies. Besides, differences in the inclusion criteria, exclusion criteria, as well as retreatment criteria might explain why our results differed from other studies.

Actually, the purpose of introducing the “1 + PRN” treatment regime to clinical use in China was to reduce economic burdens for PCV patients. Therefore, we performed the cost-benefit analysis in each group by comparing the money which was spent for improving per 0.1 logMAR units as well as the money spent for reducing per 100 μm CFT. We could see from the Table 2 that IVR treatment cost the least money (2319.53 US dollars) for gaining per 0.1 logMAR units among the three groups and the combination therapy needed the least money (2749.28 US dollars) for reducing per 100 μm of CFT among the three groups, which should be an important considering factor for the ophthalmologists when choosing an appropriate treatment regime for the patient.

The present study has several limitations: The first one is the relatively small number of patients, which results in a relatively low statistical power for the study. This is also one of the reasons why our results differed from other studies. Besides, although we have the FFA/ICGA data of the baseline and final visit time-point for all the patients, it was difficult to obtain the full follow-up FFA/ICGA data at other time-point for every patient because FA/ICGA were used at doctor’s discretion during the baseline and final visit time-point. In addition, we didn’t have data to compare the efficacy between the “1 + PRN” treatment regime and “3 + PRN” treatment regime for PCV patients in this study. However, even with these limitations, our results suggest that the “1 + PRN” treatment regime might be a favorable treatment option for macula-involved PCV patients when considering the cost-benefit especially in developing countries. However, larger, prospective, long-term, multicenter, and double-blind clinical studies are necessary to confirm the efficacy between the “1 + PRN” and “3 + PRN” treatment regime.

In conclusion, the verteporfin PDT, the IVR treatment and the combination therapy have similar efficacy in improving the BCVA as well as reducing the CFT for the macula-involved PCV patients under the “1 + PRN” treatment regimen. Cost-benefit analysis shows that IVR treatment costs the least money for improving per 0.1 logMAR units and the combination therapy demands the least money for reducing per 100 μm of CFT among three groups. Our results should provide some useful information for the ophthalmologists when choosing an appropriate treatment regime for the patients.

## Conclusion

In summary, based on our results, PDT, IVR and the combination therapy have similar efficacy in the VA improvement as well as the reduction of CFT for macula-involved PCV patients under the “1 + PRN” treatment regimen. IVR treatment costs the least money for improving per 0.1 logMAR units and the combination therapy demands the least money for reducing per 100 μm of CFT. Our results suggest that the “1 + PRN” treatment regime might be a favorable treatment option for macula-involved PCV patients when considering the cost-benefit especially in developing countries. Ophthalmologists should consider not only the treatment effect but also the cost-benefits when choosing the optimal treatment regime for the PCV patients.

## Abbreviations

AMD: Age-related macular degeneration
ANOVA: One-way repeated-measures analysis of variance
CFT: Central foveal thickness BCVA Best-corrected visual acuity
ETDRS: Early treatment diabetic retinopathy study
FFA: Fundus fluorescein angiography
ICGA: Indocyanine green angiography
IVR: Intravitreal ranibizumab injection
logMAR: Logarithm of the minimal angle of resolution
OCT: Optical coherence tomography
PCV: Polypoidal choroidal vasculopathy
PDT: Photodynamic therapy
PED: Pigment epithelial detachment
PRN: Pro re nata
RPE: Retinal pigment epithelium
VA: Visual acuity
VEGF: Vascular endothelial growth factor
